# Features of Metabolites and Biomarkers in Inflammatory and Infectious Complications of Childhood Cancers

**DOI:** 10.3390/biomedicines12092101

**Published:** 2024-09-14

**Authors:** Maria Getsina, Ekaterina Chernevskaya, Natalia Beloborodova, Evgeniy Golovnya, Petr Polyakov, Nicolai Kushlinskii

**Affiliations:** 1 Federal Research and Clinical Center of Intensive Care Medicine and Rehabilitology, Petrovka Str., 25-2, 107031 Moscow, Russia; echernevskaya@fnkcrr.ru (E.C.);; 2 Federal State Budgetary Institution «N.N. Blokhin National Medical Research Center of Oncology» of the Ministry of Health of the Russian Federation, 115522 Moscow, Russia

**Keywords:** SIRS, sepsis, aromatic microbial metabolites, presepsin, pediatric malignant oncological diseases, leukemia

## Abstract

**Background**: In the treatment of oncological diseases in children, the search for opportunities for the earlier detection of complications to improve treatment results is very important. Metabolomic studies are actively conducted to stratify different groups of patients in order to identify the most promising markers. **Methods**: Three groups of patients participated in this study: healthy children as a control group (n = 18), children with various malignant oncological diseases (leukemia, lymphoma, nephroblastoma, ependymoma, etc.) as patients (n = 40) without complications, and patients (n = 31) with complications (inflammatory and infectious). The mitochondrial metabolites (succinic and fumaric acids); biomarkers related to inflammation such as C-reactive protein (CRP), procalcitonin (PCT), and presepsin (PSP); and sepsis-associated aromatic metabolites, such as phenyllactic (PhLA), hydroxyphenyllactic (*p*-HPhLA), and hydroxyphenylacetic acids (*p*-HPhAA), were identified. **Results**: It was found that children with malignant oncological diseases had profound metabolic dysfunction compared to healthy children, regardless of the presence of systemic inflammatory response syndrome (SIRS) or sepsis. The prognostic ability of procalcitonin and presepsin for detecting sepsis was high: AUROC = 0.875, cut-off value (Youden index) = 0.913 ng/mL, and AUROC = 0.774, with cut-off value (Youden index) of 526 pg/mL, respectively. **Conclusions**: A significant increase in aromatic microbial metabolites and biomarkers in non-survivor patients that is registered already in the first days of the development of complications indicates the appropriateness of assessing metabolic dysfunction for its timely targeted correction.

## 1. Introduction

According to statistics, cancer is predominantly a disease that affects adults; the diagnosis is much less common among children [1,2]. The incidence of the disease in the European part of Russia corresponds to its value in Europe. In total, approximately 4.5 thousand children fall ill with cancer in the Russian Federation each year [3]. This is 15 children per 100 thousand children [4]. Childhood cancers differ from adult cancers in their localization [5]. According to the National Cancer Institute, oncological diseases such as leukemia, tumors of the brain or spinal cord, and lymphomas are more common in children than in adults [6]. The majority are tumors of the blood; 25% are tumors of the central or peripheral nervous system; and 25% are tumors of bones and soft tissues [7].

Conventional chemotherapy is crucial for treating pediatric cancer, particularly in low- and middle-income countries [8]. Increasing the dose of anticancer drugs often leads to the development of a wide range of adverse reactions and an increase in life-threatening complications [9]. Carrying out combined and complex methods of special treatment is also associated with the development of complications, which often determine an unfavorable prognosis of the disease. These complications include acute endotoxemia, sepsis, septic shock, and multiple organ failure. According to the reported statistics, sepsis and septic shock account for 30% of all oncological admissions to the pediatric intensive care unit (ICU) [10,11,12]. Herewith, the mortality among children with cancer is unfortunately high, and, unlike in adult cancer, it has remained relatively unchanged over the past decades [13]. The frequency of infectious complications and sepsis varies depending on the oncological diagnosis and the presence (of a combination) of risk factors, such as chemotherapy regimens, the use of immunosuppressive drugs with a high risk of neutropenia, impaired barrier function of mucous membranes, and translocation of endogenous bacteria [14]. Diagnosis is difficult due to the lack of specific diagnostic tests and depends on the identification of changes in vital signs and various systemic manifestations associated with infections that may resemble the features of various critical illnesses [15]. In 2016, the Third International Consensus Definitions for Sepsis and Septic Shock (Sepsis-3) defined sepsis as life-threatening organ dysfunction caused by a dysregulated host response to infection, in which organ dysfunction can be represented by an increase in the Sepsis-related Organ Failure Assessment (SOFA) scale of two points or more [16]. This scale apply only to adult patients and do not apply to organ dysfunction in children. Only in 2024, the Phoenix Sepsis Score were derived and validated by the international SCCM Pediatric Sepsis Definition Task Force, who recommended a score of at least two, identifying potentially life-threatening organ dysfunction in children younger than 18 years with infection [17]. There are still a number of limitations in the use of the pediatric score, and despite the exclusion of the SIRS concept from the adult Sepsis-3 algorithm due to its low specificity, the assessment of the inflammatory response is still used in everyday pediatric clinical practice [18]. We still need not only clinical criteria but also biomarkers that include a large number of inflammatory mediators and microbial products that reveal the etiopathogenetic mechanisms of sepsis. According to one review, more than 178 biomarkers of sepsis have been studied and described in 3370 scientific publications over the past few years [19].

Biomarkers are used not only as indicators of the presence or absence of sepsis but also for monitoring the effectiveness of therapy, the prognosis of the development of complications, and the outcome of the disease [20]. Unfortunately, no biomarker shows sufficient sensitivity and specificity for the early diagnosis and prognosis of sepsis in the ICU.

Metabolomic profiling is widely used to stratify different groups of patients and to identify the most promising disease markers [21,22]. The modern equipment and various methods used to study the profile of metabolites most often include nuclear magnetic resonance spectroscopy (NMR) and mass spectrometry (MS) in combination with gas chromatography or high-performance liquid chromatography (HPLC). There are a limited number of studies aimed at identifying the potential metabolomic biomarkers of sepsis [23]. Sepsis has previously been shown to result in the increased metabolism of aromatic amino acids and their metabolites [24,25]. Based on our previous studies, we hypothesized that mitochondrial and aromatic microbial metabolites may be helpful in identifying sepsis as a manifestation of the most severe metabolic dysfunction. The aim of this study was to evaluate the diagnostic value of certain laboratory parameters, such as biomarkers of inflammation, as well as mitochondrial and aromatic microbial metabolites, in children with or without infection complications during the treatment of malignant oncological diseases.

## 2. Materials and Methods

This prospective observational study was performed at the N.N. Blokhin National Medical Research Cancer Center of the Ministry of Health of the Russian Federation, Moscow. This study was approved by the local ethics committee (Protocol No. 5/21/3/Suppl. 1 dated 23 December 2021). This study was conducted in accordance with the ethical standards of the Helsinki Declaration. Consent to participate in this study was obtained from each patient or their official representative.

### 2.1. Study Design

**Figure 1:**
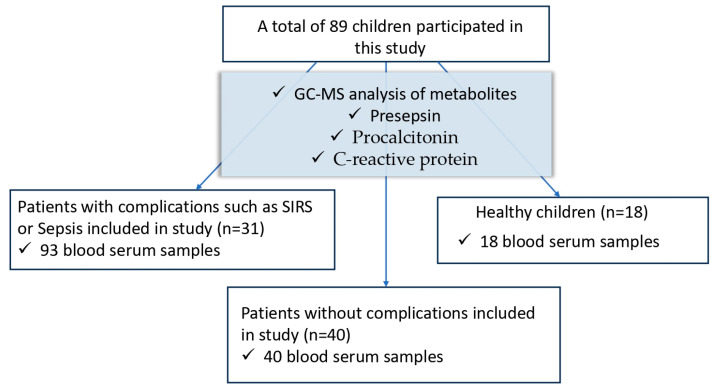
Patient groups. This study included 31 pediatric cancer patients with complications. Blood serum was collected on the first day of detection of complications—point 1, on days 2–4 of observation—point 2, and on days 5–9 of observation—point 3; a total of 93 blood serum samples were collected. The group of practically healthy children without cancer and/or infectious complications included 18 healthy children; the other group included 40 primary patients without complications.

This prospective study included pediatric patients with cancer who subsequently developed SIRS or septic complications as a result of anticancer treatment. Residual serum from two groups of patients was used as a control: practically healthy children sent for a routine examination and primary patients admitted to the clinic due to cancer. The patient groups are shown in Figure 1.

The inclusion criteria were as follows:

- Patients less than 18 years old.
- Pediatric patients with cancer.
- Risk of infectious complications (immunosuppression, neutropenia, surgical interventions).

The exclusion criteria were as follows:

- Patients over 18 years old.
- No risk of infectious complications according to clinical and laboratory data.
- Terminal stage.

### 2.2. Patients and Samples

This study included 31 pediatric patients with cancer: 19 boys and 12 girls. This group included patients from the main study group with complications of SIRS, sepsis, and septic shock. The median age was 9 years (range 6 months to 17 years). In the structure of diseases, hemoblastoses predominated—68% (leukemia, lymphoma n = 21); 32% had other oncological diseases (nephroblastoma, ependymoma, atypical terato-rhabdoid tumor, etc., n = 10). The control group included 18 practically healthy children without cancer and/or infectious complications (9 boys and 9 girls). The median age was 13.5 years. The other group included 40 primary patients under 18 years of age with confirmed cancer but without infectious and inflammatory complications (26 boys, 14 girls). The median age was 9 years. The patient characteristics are described in Table 1.

Blood samples were collected in vacuum tubes with EDTA for the determination of presepsin (PSP) in the plasma and in tubes with a coagulation activator for the determination of C-reactive protein (CRP), procalcitonin (PCT), and circulating metabolites: sepsis-associated aromatic microbial metabolites (AMMs) and mitochondrial metabolites. The blood samples were taken at three time intervals: point 1—the moment of the onset of infectious complications according to clinical data (leukocytosis/leukopenia, fever, increase in CRP in the biochemical blood test); point 2—2–4 days from the onset of infectious complications; point 3—days 5–9 of monitoring the patient’s condition. To form a group of healthy children, residual serum from patients undergoing routine medical examination was used. The samples were aliquoted and stored at −30 °C.

### 2.3. Methods

The following metabolites and biomarkers were identified: sepsis-associated metabolites: phenyllactic (PhLA), hydroxyphenyllactic (*p*-HPhLA), and hydroxyphenylacetic acid (*p*-HPhAA); mitochondrial metabolites: succinic and fumaric acids; and biomarkers: CRP, PCT, and PSP. Three groups participated in this study: a normal control group (healthy children) (n = 18); a pathological control group (patients without complications), n = 40; and patients with complications (n = 31).

#### 2.3.1. Reagents for GC–MS Analysis

3-Phenyllactic acid, CAS No. 828-01-3 (PhLA, ≥98%); 2-(4-hydroxyphenyl)acetic acid, CAS No. 156-38-7 (*p*-HPhAA, ≥98%); 3-(4-hydroxyphenyl)lactic acid, CAS No. 306-23-0 (*p*-HPhLA, ≥97%); succinic acid, CAS No. 110-15-6 (≥99%); fumaric acid, CAS No. 110-17-8 (≥99%); 2,3,4,5,6-D5-benzoic acid, CAS No. 1079-02-3 (surrogate internal standard, ≥99 atom % D, ≥99%); 3,4-dihydroxybenzoic acid, CAS No. 99-50-3 (surrogate internal standard); N,O-bis(trimethylsilyl)trifluoroacetamide, CAS No. 25561-30-2 (99%, contains 1%trimethylchlorosilane); and hexane, CAS No. 110-54-3 (≥97.0%) were obtained from Merck (Darmstadt, Germany). The sulfuric acid, CAS No. 7664-93-9; acetone, CAS No. 67-64-1; diethyl ether, CAS No. 60-29-7; and sodium chloride, CAS No. 7647-14-5 were of laboratory reagent grade and were obtained from Khimreactiv (Kemerovo, Russian Federation).

#### 2.3.2. GC–MS Analysis

A total of 151 blood serum samples were examined using the GC–MS method. The samples were defrosted at room temperature prior to use. All the GC–MS analyses were performed on a GC-2010 Plus gas chromatograph equipped with an GCMS-QP2020 mass spectrometer using the capillary column SH-5ms (95% poly(dimethylsiloxane) + 5% phenyl polysilphenylene-siloxane phase, 30 m × 0.25 mm, df = 0.25 µm) obtained from Shimadzu (Shimadzu Corporation, Tokyo, Japan). The conditions of the liquid–liquid extraction of the phenyl carboxylic acids, chromatographic separation, and mass spectrometric analysis were as previously described [26]. An internal standard (100 µL) was added to an aliquot of blood serum (200 µL), and distilled water (700 µL) was added. After extraction with diethyl ether, derivatization was performed with N,O-bis(trimethylsilyl)trifluoroacetamide (20 µL, 80 °C, 15 min). The solution with trimethylsilyl derivatives was cooled at 5 °C for 30 min and diluted with 400 µL of n-hexane, and 2 µL of the final solution was injected into the GC–MS system. The mass spectrometric analysis was performed in the total ion current (TIC) mode with electron energy 70 eV, interface temperature 250 °C, ionization chamber temperature 200 °C, *m*/*z* 50–450, scan rate 3 scans/s, cathode delay time 4 min, and cathode turn-off time 20 min. The trimethylsilyl derivatives of the phenyl carboxylic, succinic, and fumaric acids were identified using retention times and characteristic *m*/*z* values that were previously described by the instrument software (GCMS Real Time Analisis; GCMS Postrun; MS Search v.2.4). The concentrations of the phenyl carboxylic, succinic, and fumaric acids were calculated using the equations of linear functions. The linearity of the calibrations was observed at concentrations ranging from 0.5 to 90 µmol/L. The limit of quantitation for all the metabolites was 0.5 µmol/L.

#### 2.3.3. Biomarker Analysis

The CRP was determined using a Cobas 6000 biochemical analyzer (Cobas 6000, Roche Diagnostics, Risch-Rotkreuz, Switzerland). The PSP was determined on a Pathfast immunochemiluminescent analyzer (Mitsubishi Chemical Medience Corporation, Tokyo, Japan) using a standard set of reagents. The determination of the PCT was carried out using the immunochemical method with electrochemiluminescence (Cobas e411, Roche Diagnostics, Switzerland) according to the attached instructions and a set of reagents.

#### 2.3.4. Statistical Analysis

The data distribution was assessed using the Lilliefors test. Due to the significant deviation of the quantitative variables from a normal distribution, non-parametric statistical tests were used. The continuous variables were reported as medians (Mes) and interquartile ranges (IQRs), and the categorical variables were expressed as frequencies and percentages. For continuous independent variables, the Mann–Whitney U test (Wilcoxon rank-sum test) was employed. When there were more than two comparable samples, the Kruskal–Wallis test with Dunn–Bonferroni post hoc analysis was used. The Wilcoxon signed-rank test was applied for related samples, and if there were more than two samples, the Friedman test with post hoc analyses for pairwise comparisons was used. The chi-square test and Fisher’s exact test were applied for comparing categorical variables, with the Bonferroni correction used when appropriate. To determine the prognostic quality of quantitative predictors, ROC analysis was performed. The strength of the association between the parameters was evaluated using Spearman’s rank correlation coefficient. Statistical significance was set at *p* < 0.05 (two sided). All the statistical calculations were performed using IBM SPSS Statistics v. 27.0.

## 3. Results

### 3.1. Metabolic Changes

#### 3.1.1. Metabolites and Biomarkers in Healthy Children, Patients without Complications, and Patients with Complications (SIRS, Sepsis, Septic Shock)

A comparison of biomarkers in the three groups showed statistically significant differences between the groups of patients with complications and the group of healthy children and the group of patients with a diagnosis but without complications (Table 2). At the same time, a pairwise comparison of each group revealed that statistically significant differences were present between the groups of healthy donors and patients with complications, as well as between the groups with oncology without complications and with complications. When comparing the group of healthy children and group of patients without complications, no significant differences in biomarker values were observed. Patients who developed complications during treatment had statistically significant differences in the values of biomarkers such as PSP, PCT, and CRP in comparison with both healthy children and patients without complications.

It should be noted that the values for succinic acid were significantly lower in patients with complications than in healthy donors (*p*-Value < 0.05). Succinic acid was statistically significantly lower in the group of patients with complications (*p* < 0.001). When assessing the amounts of sepsis-associated acids: PhLA, *p*-HPhAA, and *p*-HPhLA, when comparing healthy children and patients with complications, no statistically significant differences were found, and in patients with oncology but without complications, the values of both *p*-HPhLA alone and the amount of Σ3AMM were statistically significantly lower.

#### 3.1.2. Monitoring Metabolites and Biomarkers in Patients with Complications

Blood serum for the analysis of metabolites and the biomarkers CRP, PCT, and PSP was collected on the first day of detection of complications—point 1, on days 2–4 of observation—point 2, and on days 5–9 of observation—point 3. The severity of the condition on the SOFA score was 4 (IQR: 4; 6), and the multiple organ failure (MOF) score was 2 (IQR: 1; 3). Table 3 presents the observation results.

Table 3 shows that the values of the biomarkers decreased statistically significantly at the third observation point. The metabolites did not show statistically significant differences between the values on the first day of complications (point 1) and on days 2–4 (point 2), as well as on days 5–9 (point 3). The correlation analysis showed that there was a positive correlation between *p*-HPhLA and PhLA throughout the entire duration of this study: the Spearman coefficient for point 1 was 0.691 (*p*-Value < 0.001); for point 2, it was 0.577 (*p*-Value < 0.001); and for point 3, it was 0.723 (*p*-Value < 0.001). A positive correlation was observed between presepsin and PhLA, throughout the entire duration of this study: the Spearman coefficient for point 1 was 0.463 (*p*-Value = 0.009); for point 2, it was 0.383 (*p*-Value = 0.033); and for point 3, it was 0.473 (*p*-Value = 0.047). The same correlation was found between PSP and PCT, throughout the entire duration of this study: the Spearman coefficient for point 1 was 0.595 (*p*-Value = 0.002); for point 2, it was 0.677 (*p*-Value < 0.001); and for point 3, it was 0.836 (*p*-Value < 0.001).

#### 3.1.3. Changes in the Metabolomic Profile in Patients with Complications

In this section, the patients were divided into groups: with complications without infection (SIRS) and patients with infectious complications (sepsis/septic shock). 

Inflammation (SIRS) was diagnosed when at least two of the following criteria were present without organ dysfunction and infection:

- Body temperature ≥ 38 °C (febrile) or ≤36 °C (hypothermia).
- Heart rate ≥ 90/min (tachycardia).
- Tachypnea: respiratory rate ≥ 20/min or hyperventilation with blood carbon dioxide ≤ 32 mmHg.
- Leukocytosis (≥12,000/μL) or leukopenia (≤4000/μL) or shift in the leukocyte formula to the left.

Sepsis was diagnosed in the presence of organ dysfunction, with an increase in the SOFA score by two points or more and suspicion or confirmation of infection. When the patients were divided into groups, the corresponding SOFA score was used as a criterion, which for the group of patients with sepsis and septic shock corresponded to 4 (IRQs: 4; 6) points and for the SIRS group to 0 (IQR: 0; 0) points. MOF in the group with sepsis and septic shock corresponded to values of 2 (IQR: 1; 3), while, in the group with complications without infections, the number of affected organs was 1 (IQR: 1; 1) point. 

Statistical analysis was carried out in these groups at point 1—the first day of complications. Table 4 shows that the metabolites did not show statistically significant differences in the groups with complications without infection (SIRS) and in patients with infectious complications (sepsis/septic shock). The values of procalcitonin and presepsin were statistically significantly greater in the group with infectious complications.

#### 3.1.4. ROC Analysis

**Figure 2:**
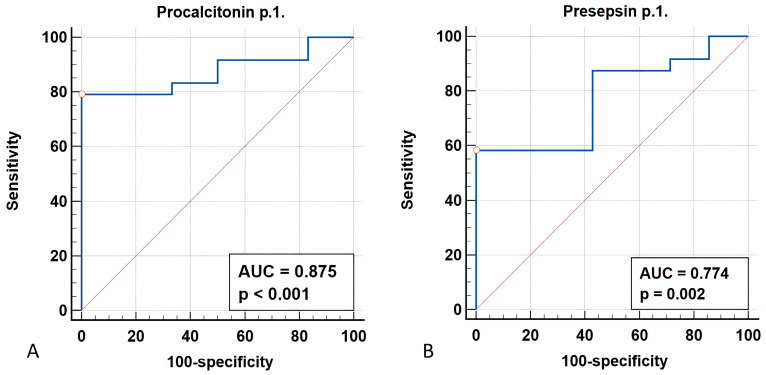
AUROC curve for procalcitonin (**A**) and presepsin (**B**) levels at time point 1.

The prognostic ability of procalcitonin and presepsin for detecting severe infectious complications, specifically sepsis and septic shock, was assessed using ROC analysis (Figure 2). Elevated procalcitonin at time point 1 was the statistically significant risk factor with high predictive power (Figure 2; *p*-Value < 0.001; AUROC = 0.875 (95% CI: 0.751–0.999); cut-off value (Youden index) = 0.913 (95% CI: 0.880–0.913), with specificity = 100% (95% CI: 54.1–100.0) and sensitivity = 79.2% (95% CI: 57.8–92.9). Also, the elevated presepsin level at time point 1 was a statistically significant risk factor with good predictive value (*p*-Value = 0.002; AUROC = 0.774, 95% CI: 0.598–0.949). A cut-off value (Youden index) of 526 pg/mL (95% CI: 316–526) was identified, with a specificity of 100% (95% CI: 59.0–100.0) and a sensitivity of 58.33% (95% CI: 36.6–77.9). No statistically significant differences in the concentrations of sepsis-associated metabolites were observed between the groups.

#### 3.1.5. Metabolomic Profile Changes in Survivors vs. Non-Survivors

Among the patients with complications (n = 31), two groups were identified: survivors (n = 28) and non-survivors (n = 3). The SOFA score in the group of those who did not survive was more than six points, and the MOF score was more than three points. Table 5 shows the results of the statistical analysis of the comparison of these groups according to the concentrations of metabolites and biomarkers on the first day of complications.

Despite the small group of deceased patients, which is a limitation for drawing indisputable conclusions, statistically significant differences were observed for ∑3AMM, *p*-HPhLA, CRP, and PSP. Succinic and fumaric acids did not show statistically significant differences. In addition, it should be noted that PhLA and *p*-HPhAA in the group of the deceased were significantly greater. But we cannot talk about statistically significant differences, since, in the group of survivors, the values of these acids were below the limit of quantification of the concentration of 0.5 µmol/L.

## 4. Discussion

### 4.1. Metabolites

Metabolomic profiling is a powerful modern tool for identifying new biomarkers and indicators of normal or pathological processes in the body. Most of these compounds are aromatic α-amino acids, tyrosine, phenylalanine, and tryptophan or metabolites of these acids, which are used for the synthesis of proteins or various biologically active compounds such as neurotransmitters and hormones that are necessary to maintain normal biological functions [27]. In addition, there are a number of aromatic metabolites that are known to be microbial metabolites of these amino acids [20]. Disruption of the microbiota is an important risk factor, leading to imbalance at the microbiome level and affecting the metabolomic profile [28,29,30]. Previously, it was shown that a change in the serum concentration of some aromatic microbial metabolites (AMMs) makes it possible to judge the violation of microbiota metabolism. For example, in patients diagnosed with pancreatic cancer before surgery, the level of some microbial and mitochondrial metabolites is lower than that in healthy volunteers [31]. We described such changes as “less and mess” (low quantity and altered composition), characterized by pronounced disturbances in the taxonomic composition of the microbiota in patients with chronic critical illness [32]. A similar pattern was observed in our study, in which the concentration of AMM in the blood of cancer patients was statistically significantly lower than that in healthy children. The data obtained in this article indicate that sepsis-associated aromatic and mitochondrial metabolites also did not change when comparing the inflammation and sepsis groups, and they did not change during treatment in patients with complications, which indicates a more extensive metabolic dysfunction in childhood cancer. However, the metabolomic approach may be useful for the differential diagnosis of bacterial sepsis. There is a difference in the metabolome between children with postoperative inflammation but without infection and those with infection (bacterial and viral), but specific metabolites were not identified [33]. A metabolomic approach was used to identify patients with bacterial sepsis in comparison with the control group. Six metabolites were identified, and myristic acid was especially notable [34]. Among amino acids, it has been noted that, during sepsis, kynurenine changes its concentration [35,36]. The concentration of tyrosine and tryptophan is statistically significantly lower in patients with SIRS and sepsis compared to that in donors. But, at the same time, there is no statistically significant difference between SIRS and sepsis [37]. The concentrations of sulfur-containing amino acids, especially taurine, decreased significantly as the severity of sepsis increased [37]. Several pathologies leading to sepsis have been identified, and a study noted that the set of metabolites that are predictors of the development of sepsis may depend on the diagnosis. Metabolome damage occurs in different ways, for example: a significant difference in phenylalanine levels was observed between acute kidney injury groups (increased concentration compared to control) and decreased phenylalanine concentration in the case of acute liver ischemia [38]. Previously, it was shown that aromatic metabolites can predict the outcome and severity of the condition in adult ICU patients but did not differentiate SIRS from sepsis/septic shock at an early stage [39].

Many studies note that, during sepsis, mitochondrial dysfunction develops, which leads to changes in the concentrations of a number of metabolites associated with mitochondria and involved in the Krebs cycle. The authors of [35,36] proposed two of these metabolites, acylcarnitine C10:1 and glycerophospholipid PCaaC32:0, for differentiating systemic inflammatory response syndrome (SIRS) and severe sepsis. With sepsis, as a result of mitochondrial dysfunction, the level of metabolites of the glycolytic cycle and the tricarboxylic acid cycle, such as lactic, pyruvic, and citric acids, increases in the circulating blood [40]. Some publications report that patients with sepsis have increased levels of glycolytic metabolites, including lactate, pyruvate [33,34,41], and succinate [41], compared to patients with inflammation, which may indicate a shift in the overall metabolism or mitochondrial dysfunction. This work noted a decrease in the values of succinic and fumaric acids when comparing cancer patients with a healthy group, which also indicates mitochondrial dysfunction. However, the difference in the content of succinic and fumaric acids in the serum of patients was not statistically significant when comparing groups with inflammation and sepsis.

The existing sepsis scoring systems are not effective in predicting attributable mortality in children with cancer who are admitted to the ICU with suspected sepsis [42]. However, despite the small number of deceased patients, statistically significant differences were observed for the metabolites ∑3AMM and *p*-HPhLA and for the biomarkers CRP and PSP.

### 4.2. Biomarkers

Several protein biomarkers have already been proposed as prognostic markers of sepsis, such as CRP, lipopolysaccharide binding protein, PCT, or cytokines such as tumor necrosis factor-α and interleukin-6 [43,44]. The concentration of PSP accurately reflects the degree of the inflammatory response and changes rapidly depending on the effectiveness of antibacterial therapy [45]. PSP is increased in patients with neutropenia, but this was only useful for predicting a poor outcome 48 h after the onset of fever [46]. The importance of PSP is noted in differentiating the state of sepsis, severe sepsis, and septic shock, in contrast to other biomarkers.

A comparison of biomarkers of systemic inflammation (PCT and CRP) and infectious lesions revealed that only the rate of change in the PSP concentration was significantly different between the group of survivors and those who died. Presepsin can be recommended for use as an early marker of sepsis in patients with cancer [46,47,48]. The present study shows that PCT and PSP not only reflect the difference between healthy donors and patients with complications, but they can also differentiate inflammation and sepsis. The work [49] showed a particularly similar threshold value of presepsin for distinguishing sepsis from inflammation as a 407 pg/mL and a significant difference was noted between Gram-positive and Gram-negative bacterial infections. However, it did not indicate what diagnoses the patients were admitted with. Other studies have shown that presepsin levels increase in sepsis regardless of the type of bacteremia [50], including in patients with acute leukemia and febrile neutropenia receiving chemotherapy [51]. Also of interest is the positive correlation that was observed between PSP and PhLA throughout this study. The production of PhLA is the one that is most expressed by the microbiota of septic patients, and it is associated with the Enterobacteriaceae members [28].

Our study has a number of significant limitations: the number of patients included in this study was small; the sample was rather heterogeneous; and the patients differed in diagnoses, stages of cancer, the presence of concomitant diseases, treatment, etc. However, in conclusion, it should be noted that, in this pilot project, significant changes in the metabolic profile in pediatric cancer were revealed when compared with healthy children. The absence of differences in the metabolic profile when comparing the inflammation and sepsis groups indicate profound disorders in the metabolic pathways associated, probably, with the main malignant disease, and it is extremely important to establish causal relationships in the future. The results of our study indicate the prospects for broader metabolomic research in pediatric oncology.

## 5. Conclusions

Our study highlights profound metabolic dysfunction in childhood malignancies, as the serum mitochondrial metabolites and aromatic amino acid metabolites significantly differed in the examined children compared to healthy children. Effective therapy significantly reduced the concentrations of inflammatory biomarkers (CRP, PCT, PSP) but did not normalize the concentrations of aromatic microbial metabolites in patients with complications, which presumably indicates the lack of targeted therapy aimed at modulating mitochondrial and microbiome metabolism. A statistically significant increase in the concentrations of sepsis-associated aromatic microbial metabolites (*p*-HPhLA, Σ3AMM) and biomarkers (CRP, PSP) in non-survivors suggests that metabolic dysfunction should be assessed for its timely targeted correction in the future. This study also identified the high prognostic ability of procalcitonin and presepsin for detecting sepsis: AUROC = 0.875; cut-off value (Youden index) = 0.913 ng/mL, and AUROC = 0.774, with cut-off value (Youden index) of 526 pg/mL, respectively.

## Figures and Tables

**Table 1:** Characteristics of healthy children (n = 18), patients with oncology without complications (n = 40), and patients in the main study group with complications (SIRS, sepsis, and septic shock) (n = 31). Age data are presented as medians (Mes) and interquartile ranges (IQRs). The distribution of patients by cancer stage is described, n (%) as is the number of patients with a diagnosis of leukemia, n (%) and the number of bone marrow transplants, n (%). The systemic pathologies present, n (%), and the complications that resulted from treatment, n (%) are also described. Each patient may have had several systemic pathologies.

Parameters	Healthy Children (n = 18)	Patients without Complications (n = 40)	Patients with Complications (n = 31)
Gender, males, n (%)	9 (50.0%)	26 (65.0%)	19 (61.3%)
Gender, females, n (%)	9 (50.0%)	14 (35.0%)	12 (38.7%)
Age, years (Me (IQR))	13.5 (8.0; 16.0)	9.0 (4.0; 12.0)	9.0 (4.0; 15.0)
Cancer stage, n (%)	-	I-8 (20.0%) II-10 (25.0%) III-9 (22.5%) IV-13 (32.5%)	I-9 (29.0%) II-12 (38.7%) III-5 (16.1%) IV-5 (16.1%)
Bone marrow transplantation, n (%)	-	1 (2.5%)	2 (6.5%)
Leukemia, n (%)	-	12 (30.0%)	14 (45.0%)
Systemic pathology, n (%)	-	-	Aplasia—15 (48.4%), cardiovascular system—8 (25.8%), digestive—8 (25.8%), respiratory organs—7 (22.6%), kidneys—5 (16.1%), CNS—3 (9.7%), SPON—1 (3.2%), liver—1 (3.2%)
Complications after treatment, n (%)	-	-	Enterocolitis—8 (25.8%), coagulopathy—5 (16.1%), mucositis—4 (12.9%), stomatitis—4 (12.9%), pleurisy—2 (6.5%), bronchitis—2 (6.5%), hemorrhagic syndrome—1 (3.2%), shock—1 (3.2%), epilepsy—1 (3.2%), pyoderma—1 (3.2%)

**Table 2:** Metabolites and biomarkers in healthy children (n = 18), patients with oncology without complications (n = 40), and patients with complications (SIRS, sepsis, and septic shock) (n = 31).

Metabolites, μmol/L/Biomarkers	Healthy Children (n = 18)	Patients without Complications (n = 40)	Patients with Complications (n = 31)	*p*-Value
PhLA	<0.5 (<0.5; <0.5)	<0.5 (<0.5; <0.5)	<0.5 (<0.5; 0.9)	-
*p*-HPhAA	<0.5 (<0.5; <0.5)	<0.5 (<0.5; 0.6)	<0.5 (<0.5; 1.0)	-
*p*-HPhLA	1.4 (1.1; 1.8)	0.9 (0.7; 1.1)	1.1 (0.8; 1.7)	<0.001
Σ3AMM	2.2 (1.5; 2.6)	1.5 (1.1; 1.8)	2.0 (1.6; 3.3)	0.001
Succinic acid	3.4 (2.7; 4.8)	2.8 (2.5; 3.6)	1.9 (1.3; 3.1)	<0.001
Fumaric acid	1.1 (0.9; 1.2)	0.7 (0.6; 0.9)	0.8 (0.4; 1.2)	0.002
CRP (mg/L)	0.3 (0.2; 3.8)	1.35 (0.9; 2.4)	128 (66; 201)	<0.001
PCT (ng/mL)	0.06 (0.03; 0.07)	0.07 (0.05; 0.12)	4.2 (0.6; 11.8)	<0.001
PSP (pg/mL)	93 (72; 111)	108 (91; 122)	526 (331; 917)	<0.001

**Table 3:** Metabolites and biomarkers in patients with complications (SIRS, sepsis, and septic shock) (n = 31) over time: on the first day of detection of complications—point 1, on days 2–4 of observation—point 2, and on days 5–9 of observation—point 3.

Metabolites, μmol/L/Biomarkers	Patient Point 1 (n = 31)	Patient Point 2 (n = 31)	Patient Point 3 (n = 31)	*p*-Value
PhLA	<0.5 (<0.5; 0.9)	<0.5 (<0.5; 0.7)	<0.5 (<0.5; <0.5)	-
*p*-HPhAA	<0.5 (<0.5; 1)	<0.5 (<0.5; 0.9)	<0.5 (<0.5; 0.5)	-
*p*-HPhLA	1.1 (0.8; 1.7)	1.0 (0.8; 1.5)	1.1 (0.8; 1.4)	0.411
Succinic acid	1.9 (1.3; 3.1)	2.0 (1.2; 2.9)	1.6 (1.3; 2.7)	0.949
Fumaric acid	0.8 (<0.5; 1.2)	0.7 (<0.5; 1.3)	0.6 (0.5; 1.4)	0.841
CRP (mg/L)	128 (66; 201)	89 (40; 128)	52 (13; 98)	0.013
PCT (ng/mL)	5.24 (3; 16.2)	4.2 (1; 13.1)	2.6 (0.9; 6.8)	0.003
PSP (pg/mL)	526 (331; 917)	517 (213; 941)	483 (278; 685)	0.034

**Table 4:** Metabolites and biomarkers in patients with SIRS (n = 7) and sepsis/septic shock (n = 24) on the first day of complications.

Metabolites, μmol/L/Biomarkers	Patients with Sepsis/Septic Shock (n = 24)	Patients with Inflammation (n = 7)	*p*-Value
Point 1			
PhLA	<0.5 (<0.5; 0.9)	0.5 (<0.5; 0.7)	0.872
*p*-HPhAA	<0.5 (<0.5; 0.9)	0.6 (<0.5; 1.0)	0.216
*p*-HPhLA	1.1 (0.9; 1.7)	1.3 (0.8; 1.7)	0.729
Σ3AMM	1.7 (1.4; 3.5)	2.3 (2.1; 2.9)	0.274
Succinic acid	2.0 (1.3; 2.8)	1.8 (1.4; 3.1)	0.908
Fumaric acid	0.7 (<0.5; 1.2)	0.8 (0.5; 1.2)	0.945
CRP (mg/L)	147 (67; 202)	119 (64; 128)	0.234
PCT (ng/mL)	5.3 (3.2; 17.3)	0.45 (0.28; 0.88)	0.003
PSP (pg/mL)	662 (416; 940)	316 (179; 526)	0.029

**Table 5:** Metabolites and biomarkers on the first day of complications in patients with complications who survived (n = 28) and in non-survivors (n = 3).

Metabolites, μmol/L/Biomarkers	Patients Who Survived (n = 28)	Non-Survivor Patients (n = 3)	*p*-Value
PhLA	<0.5 (<0.5; 0.7)	1.4 (0.6; 3.7)	-
*p*-HPhAA	<0.5 (<0.5; 0.9)	1.02 (<0.5; 1.59)	-
*p*-HPhLA	1.1 (0.8; 1.4)	2.1 (1.9; 10.6)	0.007
Σ3AMM	1.9 (1.5; 2.8)	3.5 (3.5; 15.8)	0.018
Succinic acid	1.8 (1.3; 2.4)	3.1 (2.4; 3.6)	0.065
Fumaric acid	0.7 (0.4; 1.2)	1.1 (0.6; 1.7)	0.385
CRP (mg/L)	120 (65; 185)	239 (174; 317)	0.030
PCT (ng/mL)	4.1 (0.5; 11.8)	6.9 (0.7; 161)	0.663
PSP (pg/mL)	474 (324; 740)	942 (938; 2331)	0.024

## Data Availability

The original contributions presented in the study are included in the article, further inquiries can be directed to the corresponding author.
